# Stage-Stratified Analysis of Prognostic Significance of Bax-Interacting Factor-1 Expression in Resected Colorectal Cancer

**DOI:** 10.1155/2013/329839

**Published:** 2013-09-23

**Authors:** Yoon Ho Ko, Young-Seok Cho, Hye Sung Won, Ho Jung An, Der Sheng Sun, Soon Uk Hong, Jin Hee Park, Myung Ah Lee

**Affiliations:** ^1^Department of Internal Medicine, Uijeongbu St. Mary's Hospital, The Catholic University of Korea College of Medicine, Uijeongbu 480-717, Republic of Korea; ^2^Department of Pathology, Asan Medical Center, University of Ulsan College of Medicine, Seoul 138-736, Republic of Korea; ^3^Department of Biomedical Science, The Catholic University of Korea College of Medicine, Seoul 137-701, Republic of Korea; ^4^Department of Internal Medicine, Seoul St. Mary's Hospital, The Catholic University of Korea College of Medicine, Seoul 137-701, Republic of Korea

## Abstract

*Background/Aim.* Bax-interacting factor-1 (Bif-1) plays a crucial role in apoptosis and autophagy. The aim of this study was to evaluate Bif-1 protein expression and its prognostic significance in colorectal cancer (CRC). *Methods.* We analyzed Bif-1 protein expression in 251 resected specimens from patients with CRC by immunohistochemistry using tissue microarray. *Results.* Low Bif-1 expression was observed in 131 patients (52.2%) and high Bif-1 expression in 120 patients (47.8%). No significant differences were observed in clinicopathological parameters between patients with high and low Bif-1 expression. Kaplan-Meier survival analysis showed no difference in survival between patients with high and low Bif-1 expression. Stratified analysis of Bif-1 according to TNM stage demonstrated that low Bif-1 expression was significantly associated with a poor outcome in patients with stages I and II (*P* = 0.034). Stratified multivariate analysis demonstrated that low Bif-1 expression was an independent indicator of poor prognosis (hazard ratio, 0.459; 95% confidence interval, 0.285–0.739; *P* = 0.001). *Conclusion.* Patients with low levels of Bif-1 expression have shortened survival rates in CRC of stages I and II. This suggests that Bif-1 protein expression may be a useful prognostic marker in early-stage CRC.

## 1. Introduction

Colorectal cancer (CRC) is one of the leading causes of death from cancer worldwide [1]. The incidence of CRC in a number of Asian countries is rapidly increasing, causing a major health concern [2]. The adenoma-carcinoma sequence is the basis for tumorigenesis and progression of CRC, and a number of molecular changes have been identified [3]. Alterations in the expression of and mutations in apoptosis-related proteins play a key role in CRC progression and response to chemotherapy. Thus, increased understanding of changes in gene expression during colon cancer progression will contribute to clinical management and prevent development of advanced disease [4].

Bax-interacting factor-1 (Bif-1, also known as endophilin B1 and SH3GLB1), a member of the membrane curvature-driving endophilin family of proteins, associates with the proapoptotic Bcl-2 family protein Bax [5, 6] and promotes Bax conformational changes to induce apoptosis [7]. Inhibition of Bif-1 expression *in vitro* abrogates cytochrome *c* release and caspase-3 activation induced by various intrinsic apoptosis signals, and *Bif-1* knockout mouse shows delayed mitochondrial apoptosis [7]. These findings support an important role for Bif-1 in apoptotic activation, as loss of this molecule is involved in tumorigenesis.

Bif-1 also regulates the induction of autophagy [8, 9]. Autophagy, an evolutionarily conserved catabolic process, is involved in the regulation of a variety of physiological and pathological processes such as cell differentiation, immunity, energy homeostasis, cell death, and carcinogenesis [10]. During autophagy, Bif-1 interacts with beclin-1 through the ultraviolet irradiation resistance-associated gene (UVRAG) and functions as a positive regulator of the class III phosphatidylinositol-3-kinase (PI3KC3, also known as Vps 34) [8]. In addition, knockout of *Bif-1* promotes spontaneous tumorigenesis in mice [8], and allelic loss of *Bif-1* promotes chromosomal instability and accelerates the development of *Myc*-induced lymphoma by suppressing mitophagy [9]. These results suggest that Bif-1 functions as an autophagy activator and a tumor suppressor.

The human *Bif-1* gene is located on chromosome 1p22, a region that is frequently deleted in many tumor types [11]. Decreased Bif-1 expression was found in cancer cells compared to adjacent normal tissues in various human malignancies, including gastric cancer [12], prostate cancer [13], invasive bladder cancer [14], pancreatic cancer [15], and CRC [16]. However, the clinical implications of Bif-1 expression are controversial. Most studies have not demonstrated any prognostic role of reduced Bif-1 expression in several types of solid cancers. Moreover, Fan et al. reported that the protein expression of Bif-1 was significantly higher in hepatocellular carcinoma (HCC) than in the adjacent matched nontumor tissues, and its overexpression was significantly correlated with a low grade of differentiation and a shortened overall survival [17]. Although the mechanism of this discrepancy needs to be clarified, we speculate that the pathophysiological roles of Bif-1 in tumorigenesis and tumor progression may vary according to the tumor cell type due to differences in the tumor characteristics and the microenvironment of the tumor tissue.

Data correlating Bif-1 protein expression and patient survival in CRC are lacking. In this study, we analyzed the expression of Bif-1 protein in CRC tissue by immunohistochemistry using a tissue microarray and evaluated its relationship with clinicopathological parameters and survival. 

## 2. Materials and Methods

### 2.1. Patients and Specimens

All tissues were obtained from consecutive patients with CRC who had undergone surgery from January 1999 to December 2005 at Seoul St. Mary's Hospital of the Catholic University of Korea. In total, 289 cases of pathologically confirmed specimens were evaluated. Patients were excluded from this study on the basis of the following exclusion criteria: missing patient history, medical files or specimens (*n* = 4), the occurrence of surgery-related deaths (*n* = 5), the follow up <3 months (*n* = 15), and paraffin blocks not available for recutting (*n* = 14). Following these criteria, the clinical data of 251 patients were analyzed. Patient clinicopathological data were retrieved from medical records and included age, sex, histopathological diagnosis, pathological tumor stage, and patient outcomes, such as dates of death, last followup, and relapse. Histological classification was determined according to the World Health Organization (WHO) criteria, and postoperative pathological staging was analyzed according to the American Joint Committee on Cancer (AJCC) staging criteria, 7th edition. This study was approved by the Institutional Research Ethics Board of Seoul St. Mary's Hospital of the Catholic University of Korea (IRB No. KC10SIMI0621) and adhered to the Declaration of Helsinki. Patient anonymity was preserved.

### 2.2. Tissue Microarray Methods

To construct the tissue microarray block, morphologically representative tissue areas were marked on standard hematoxylin-and-eosin-stained sections that were cut from archival formalin-fixed paraffin-embedded tissue blocks; these corresponding areas were then punched out using a 2.0 mm punch, and the cores were harvested into a recipient paraffin block, with 30 cores assembled on a recipient paraffin block. To minimize any sampling error and to reduce the impact of tissue loss during processing, duplicate tissue cores for each specimen were arrayed on a second recipient paraffin block. After construction, 4 *μ*m sections were cut from the completed array block and transferred to silanized glass slides.

### 2.3. Immunohistochemistry and Analysis

Immunohistochemistry was performed on 4 *μ*m sections from the tissue microarray blocks using a Lab Vision Autostainer LV-1 (LabVision/Neomarkers, Fremont, CA, USA), according to the manufacturer's protocol. Tissue sections were mounted on superfrost glass slides, deparaffinized, and rehydrated through xylene and serial alcohol solutions. For antigen retrieval, slides were immersed in 0.01 M citrate buffer (pH 6.0) by heating the sample in a PT Link (Dako, Glostrup, Denmark) at a preheated temperature of 65°C for holding and a targeted final temperature of 95°C for 20 min. Tissue sections were treated with 0.3% hydrogen peroxide in methanol for 30 min to block endogenous peroxidase activity. A rabbit polyclonal antibody against Bif-1 was purchased from Abcam (Cambridge, UK) and was used at 1 : 10 dilution. Tissue sections were incubated with primary antibodies at room temperature for 24 h. Immunoreactions were detected by a conventional labeled streptavidin-biotin method (LSAB2 System-HRP; Dako, Carpinteria, CA, USA). The color reaction was completed by a 5 min incubation with 3,3′-diaminobenzidine, and hematoxylin counterstaining was used. For the negative controls, sections were treated using the same method with the exception that they were incubated with the antibody diluents instead of the primary antibodies. Results were analyzed by one pathologist (Soon Uk Hong), who was blinded to any clinical patient data for each case. Following scoring of samples by a pathologist, the same samples were scored repeatedly using the same procedure, but without accessing any previous data. Cases with different scores were then evaluated once more, also without knowledge of the previous results. The final scores were then entered into the database for analysis by another investigator (Yoon Ho Ko). Immunostaining was interpreted using a semiquantitative histological score. Staining intensities were classified as negative (0), weak (1+), moderate (2+), or strong (3+) staining, and the area percentage of positive staining was scored as 0 (0%), 1 (1–33%), or 2 (34%–66%), or 3 (67%–100%). A composite score was calculated by multiplying the intensity and percentage scores. Using a simple median split, that is, <2 or =2, immunoreactivity of Bif-1 expression was categorized as low or high under light microscopy.

### 2.4. Statistical Methods

Overall survival duration was calculated from the date of diagnosis to the date of death or the last follow-up visit. Cases lost to followup and deaths caused by problems other than CRC were censored during survival analysis. The influence of prognostic factors on tumor-related survival was assessed by Kaplan-Meier analysis; the log-rank test was used to calculate differences between curves. Cox proportional hazards regression models were used to investigate the significance of prognostic factors. Cumulative survival curves for patients according to Bif-1 immunoreactivity were analyzed after stratification by TNM stage. Correlations between immunohistochemical profiles and clinicopathological variables were analyzed by the *χ* ^2^ and Fisher's exact tests. Survival rates and odds ratios are presented with their respective 95% confidence intervals (CIs). A *P* value of <0.05 was considered to indicate statistical significance. Statistical analyses were performed using the SPSS software package (version 18.0; SPSS, Chicago, IL, USA).

## 3. Results

### 3.1. Patients' Clinical Characteristics

In total, 251 paraffin blocks of tumor samples were available from patients who had undergone surgery. No patient received radiation or chemotherapy preoperatively. The clinical and pathological characteristics of the series are shown in Table 1. The patient cohort consisted of 136 males and 115 females, with a median age of 63 years (range, 30−83 years). Histologically, most patients (89.6%) had tubular adenocarcinoma. According to the American Joint Committee on Cancer staging criteria, 37 patients (14.7%) had stage I disease, 60 (23.9%) had stage II, 97 (38.6%) had stage III, and 57 (22.7%) had stage IV disease. One hundred and seventeen (46.6%) patients underwent adjuvant chemotherapy and/or radiation therapy, 72 (28.7%) received 5-fluorouracil or chemotherapy, and 45 (17.9%) received 5-fluorouracil-based concurrent chemoradiation or radiation alone. The median follow-up duration was 70.1 months (range, 3.3−197.9 months) after surgical resection. Among the 251 patients, 81 (32.3%) died of their tumors, and 170 (67.7%) were alive at the last followup.

### 3.2. Bif-1 Protein Expression in CRC

**Figure 1:**
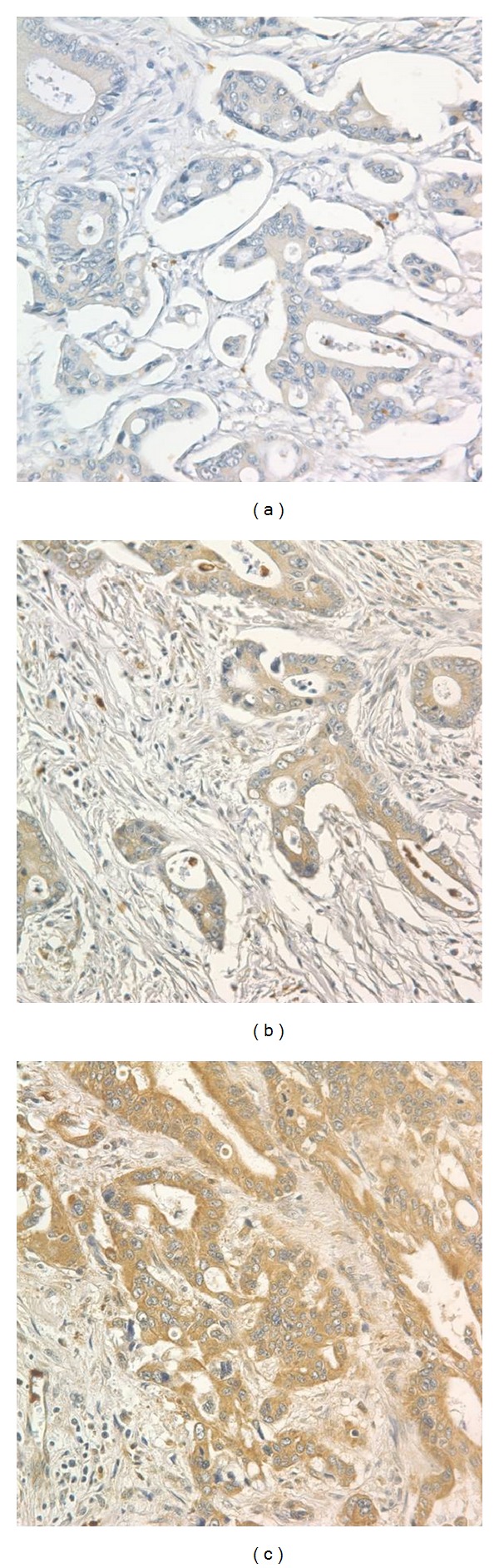
Immunohistochemical staining of Bif-1 in colorectal cancer tissues. Weak (a), moderate (b), and strong (c) expression of Bif-1. Original magnification, 200x.

Figure 1 shows representative examples of CRC stained immunohistochemically for Bif-1, along with their respective Bif-1 expression staining intensity. Of 251 patients, the intensity of staining was negative in 90 patients (35.9%), weak in 45 (17.9%), moderate in 55 (21.9%), and strong in 61 (24.3%). The area of immunoreactive tumor cells was zero in 90 patients (35.9%), <33% in 40 (15.9%), 33−66% in 43 (17.1%), and >66% in 78 (31.1%). Using a simple median split (median = 2), of 251 patients, low immunoreactivity for Bif-1 was observed in 131 patients (52.2%) and high Bif-1 expression in 120 (47.8%).

### 3.3. Correlation between Bif-1 Protein Expression and Clinicopathological Findings

The correlations between Bif-1 expression and the clinicopathological characteristics are summarized in Table 2. No significant differences were observed in age, gender, the type of histology, histological differentiation, lymphovascular invasion, depth of invasion, and lymph node metastasis between high and low Bif-1 immunostaining. However, patients with low Bif-1 expression were more likely to have a primary tumor site within the left colon and rectum than those with high Bif-1 expression (*P* = 0.034). Of the 131 patients with low Bif-1 expression, 22 (16.8%) were in stage I, 36 (27.5%) in stage II, 51 (38.9%) in stage III, and 22 (16.8%) in stage IV; of the 120 patients with high Bif-1 expression, 15 (12.5%) were in stage I, 24 (20%) in stage II, 46 (38.6%) in stage III, and 35 (29.2%) in stage IV. The proportion of low Bif-1 expression was significantly lower in stage IV cancer than in stage I, stage II, or stage III cancers (*P* = 0.022; Table 2).

### 3.4. Bif-1 Expression and Clinical Outcomes

The overall 5-year survival rate for resected CRCs was 80.9%. The median overall survival time was not yet been reached for all patients. Univariate analysis of clinicopathological factors relevant to patient survival revealed that the following factors were significant for the overall survival of patients: the type of histology (*P* = 0.002), lymphovascular invasion status (*P* < 0.001), the depth of invasion status (*P* < 0.001), lymphatic invasion status (*P* < 0.001), curative resection status (*P* < 0.001), regional lymph node metastasis status (*P* < 0.001), and TNM stages (*P* < 0.001). Kaplan-Meier analysis of the data for a total of 251 patients showed that the overall survival between patients with high versus low Bif-1 expression was not significantly different (logrank test; *P* = 0.363). To eliminate the influence of stage on prognosis, we performed a stratified analysis of Bif-1 according to TNM stage.

**Figure 2:**
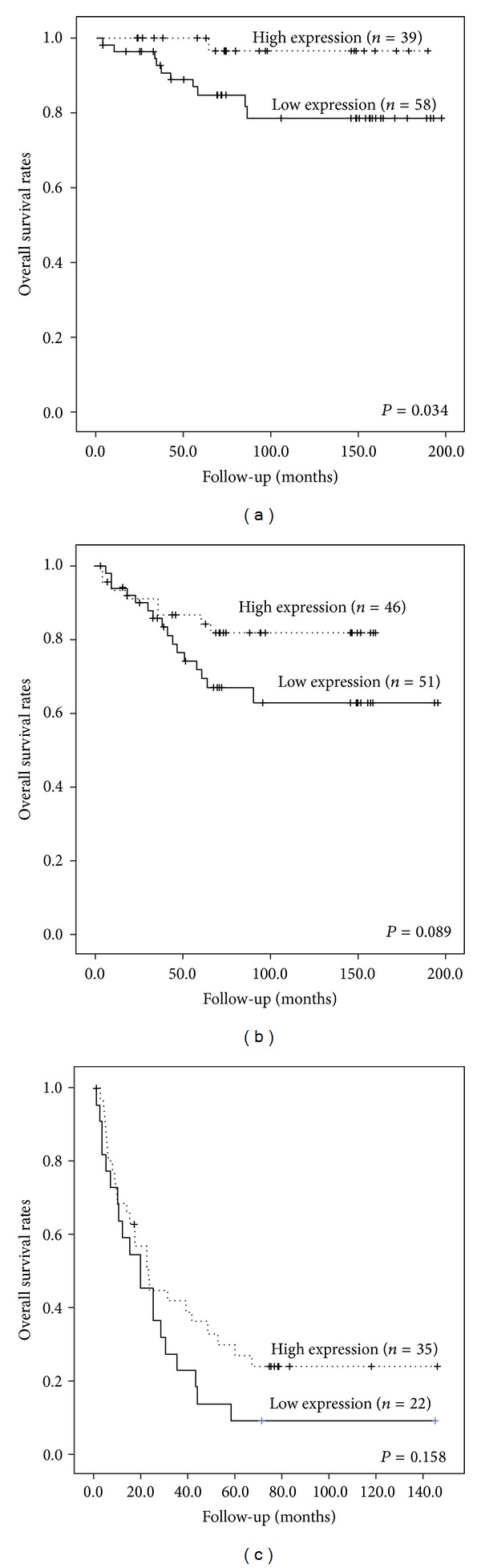
Kaplan-Meier analysis of patients with colorectal cancer according to Bif-1 expression pattern. Stages I and II (a), stage III (b), and stage IV (c).

In stages I and II patients, low Bif-1 expression resulted in a more unfavorable prognosis than those with a high Bif-1 expression; the mean overall survival time was 166.1 months (95% CI, 147.4–183.9) for low Bif-1 expression patients and 185.8 months (95% CI, 177.8–193.9) for high Bif-1 expression patients (*P* = 0.034; Figure 2(a)). In stages III and IV cancers, low levels of Bif-1 expression were associated with a nonsignificant trend toward a poorer clinical outcome (*P* = 0.089; Figure 2(b), and *P* = 0.158; Figure 2(c), resp.). A stratified multivariate analysis was performed to determine relationships between the above factors and prognosis for all patients. Curative resection status (*P* < 0.001), distant metastasis (*P* < 0.001), and regional lymph node metastasis status (*P* = 0.002) were significant poor prognostic factors. In addition, low Bif-1 expression was found to be the independent indicator of poor prognosis (Hazard ratio, 0.459; 95% CI, 0.285–0.739; *P* = 0.001; Table 3).

## 4. Discussion

Loss of Bif-1 tumor suppressor activity has been reported in a variety of tumor types and plays an important role in carcinogenesis [7–9]. However, the clinical prognostic significance of Bif-1 protein expression in CRC has not been elucidated. In the present study, we found that the low levels of Bif-1 protein expression were associated with poor clinical outcome in early stages of CRC, while higher levels of Bif-1 protein expression were observed more frequently in advanced stages of the disease. The prognostic impact appeared to be independent, even after adjusting for well-known clinicopathological parameters.

Coppola et al. reported negative Bif-1 expression in 23 of 102 CRC specimens at different stages (22.5%) and in zero of 38 samples of normal colorectal mucosa [16]. In addition, mean score of Bif-1 expression was significantly lower in CRC than in normal mucosa, and decreased Bif-1 expression in cancer cells was confirmed at the mRNA level. In other types of gastrointestinal cancer, Bif-1 protein expression by immunohistochemistry was negative in ~60% of gastric cancer [12] and 15% of gallbladder cancer [14] and was lower in 23% of pancreatic cancer cases [15]. These findings support the proapoptotic and tumor suppressive activity of Bif-1 protein.

The loss of Bif-1 protein expression in cancer cells could be interpreted functionally in several ways. Somatic mutation of *Bif-1* is rare in common human malignancies [18]. Chromosome 1p22, where the *Bif-1* is localized, has been postulated as a prominent deletion in CRC, and 1p22 deletion was significantly more common in metastatic as compared with primary CRC [19]. Another study showed that 1p22 preferentially deleted in microsatellite stable CRC [20]. These results suggest that 1p22 deletion contributes to colorectal carcinogenesis. Bif-1 could also act as a tumor suppressor due to its regulation of Bax [12]. Bif-1 accelerates Bax conformational change either through a phosphorylation-dependent mechanism or by directly binding Bax and enhances the kinetics of apoptosis induction in response to intrinsic apoptotic signals, resulting in an increase in outer mitochondrial membrane permeabilization [7]. The prognostic role of Bax protein in biological behavior and clinical prognosis in various types of cancer, including CRC, has been studied widely. Reduced expression of Bax was correlated with poor tumor differentiation, mucinous histologic type, and metastatic progression [21] and is a negative prognostic factor in patients with CRC [22, 23]. This prognostic role of Bax accords with clinical significance of Bif-1 in our study, in which low Bif-1 expression is a negative prognostic factor for overall survival. In contrast, in patients with HCC, high-intensity Bif-1 expression was correlated with a shorter survival time compared to patients with a low-intensity expression [17]. These conflicting results may be caused by the complexity of the biological functions of Bif-1.

Several pathological features, such as the depth of invasion status, tumor grade, nodal metastasis, lymphatic or vascular invasion, and curative resection status, are prognostic clinicopathological parameters in patients with CRC. We also investigated the value of Bif-1 expression as a molecular prognostic indicator and found that low levels of Bif-1 expression were an independent negative prognostic marker in stage-stratified multivariate regression analysis for disease stages I and II (mean survival, 185.8 versus 166.1 months for Bif-1 negative tumors; *P* = 0.034). The clinical value of Bif-1 expression in various types of solid cancer remains controversial. In an experimental study using a breast cancer metastatic model, Bif-1 expression was downregulated during *in situ* clinical progression of breast carcinoma to invasive and metastatic carcinoma [24]. Furthermore, expression of Bif-1 protein has not been correlated with histological characteristics and clinical outcomes in various tumors, including gastric cancer [12], invasive bladder cancer [14], pancreatic cancer [15], and CRC [16].

Another theory for the discrepancy in Bif-1 expression involves autophagy. Autophagy, type II programmed cell death, is usually activated in response to adverse environments—during which cytoplasmic materials are enclosed in double membrane-bound vesicles targeted by the lysosome for degradation [25]. Autophagy plays an important role in tumor suppression. Beclin 1, the essential autophagy regulator, is monoallelically deleted in many human ovarian, breast, and prostate cancers, and targeted mutant mice with heterozygous disruption of beclin 1 are more prone to the development of spontaneous tumors [26]. Bif-1 interacts with beclin 1 through UVRAG, and it is a positive regulator of PI3KC3, resulting in the induction of autophagy in mammalian cells [8]. In addition, Bif-1 is required for the trafficking of Atg9, transmembrane protein essential for autophagy, and the fission of Golgi membranes during the induction of autophagy [27]. Thus, the loss of Bif-1 significantly inhibits PI3KC3 activation and the formation of autophagosomes in cancer cells. Mice lacking *Bif-1* are prone to tumorigenesis, although embryonic *Bif-1* ^−/−^ mice develop normally [8]. These findings support current concept of tumor suppressive action of Bif-1 via induction of autophagy. Recently, Takahashi et al. reported enhanced accumulation of mitochondrial markers and reduced degradation of p62 in *Bif-1* ^−/−^ cells after exposure to hypoxic and low-nutrient conditions, suggesting that Bif-1-mediated mitophagy might contribute to the suppression of DNA damage during metabolic stress [9].

Our findings demonstrate increased expression of Bif-1 in advanced-stage CRC, where autophagy may be required to provide essential nutrients to the cells in the inner part of solid tumors that do not have direct access to adjacent tumor vasculature [28]. Previous study did not demonstrate any associations between Bif-1 expression and TNM stage in patients with CRC [16]. These discrepant results in CRC may be attributable—at least in part—to differences in antibodies used for staining, criteria for defining stain positivity, and patient demographics; our cohort included relatively large numbers of patients with stage IV disease (*n* = 57, 22.7%). Emerging evidence indicates that autophagy has a context-dependent role in cancer. The prosurvival role of autophagy under stressful conditions, such as hypoxia or cancer treatment, can promote tumor development [25]. Therefore, further studies are required to elucidate the precise mechanism of associations between Bif-1 expression and TNM stage in CRC.

Our findings have clear clinical implications for CRC, although the relative smaller sample sizes of each stage did not allow definite conclusions regarding the prognostic value of Bif-1 protein. Taken together, our data suggest Bif-1 expression as a new prognostic marker for early stage CRC, because patients with reduced Bif-1 expression exhibited shortened survival in stages I and II. Thus, Bif-1 expression may be a candidate biomarker for selection of adjuvant chemotherapy in stage II CRC. Further studies with a larger number of patients are required to determine the role of Bif-1 protein in early-stage CRC.

## Figures and Tables

**Table 1:** Baseline clinicopathological characteristics of patients with colorectal cancer.

Characteristics	Total	
	Patient number	%
Patient number	251	
Age (years), median (range)	63 (30–83)	
<65	115	45.8
≥65	136	54.2
Gender		
Male	136	54.2
Female	115	45.8
Histology		
Tubular adenocarcinoma	225	89.6
Others*	26	10.4
T stage		
1	2	0.8
2	49	19.5
3	154	61.4
4	46	18.3
N stage		
0	107	42.6
1	80	31.9
2	64	25.5
Stage		
I	37	14.7
II	60	23.9
III	97	38.6
IV	57	22.7
Histological grade		
Well/moderately	211	84.1
Poorly	40	15.9
Lymphovascular invasion		
Negative	92	36.7
Positive	159	63.3
Site of primary tumor		
Right colon	61	24.3
Left colon	84	33.5
Rectum	106	42.3
Curative resection (R0 resection)		
Yes	197	78.5
No	54	21.5

*Mucinous carcinoma (*n* = 25); signet-ring-cell carcinoma (*n* = 1).

**Table 2:** Relationships between clinicopathological factors and Bif-1 expression patterns.

	Bif-1		*P*
	Low, *n* (%)	High, *n* (%)	
T stage			0.839
1	1 (0.8)	1 (0.8)	
2	28 (21.4)	21 (17.5)	
3	80 (61.0)	74 (61.7)	
4	22 (16.8)	24 (20.0)	
Lymph node involvement			0.068
No	63 (48.1)	44 (36.7)	
Yes	68 (51.9)	76 (63.3)	
Distant metastasis			0.019*
No	109 (83.2)	85 (70.8)	
Yes	22 (16.8)	35 (29.2)	
Stage			0.091
I	22 (16.8)	15 (12.5)	
II	36 (27.5)	24 (20)	
III	51 (38.9)	46 (38.3)	
IV	22 (16.8)	35 (29.2)	0.022^∗†^
Site of primary tumor			0.034*
Right colon	23 (17.6)	38 (31.7)	
Left colon	48 (36.6)	36 (30.0)	
Rectum	60 (45.8)	46 (38.3)	
Histology			0.631
Tubular adenocarcinoma	117 (89.3)	108 (90)	
Others^‡^	14 (10.7)	12 (10)	
Lymphovascular invasion			0.434
Negative	51 (38.9)	41 (34.2)	
Positive	80 (61.1)	79 (65.8)	

*Statistically significant (*P* < 0.05).

^†^When pathologic stage was divided into stages I–III and IV.

^‡^Mucinous carcinoma (*n* = 25); signet-ring-cell carcinoma (*n* = 1).

**Table 3:** Predictive factors of survival by stratified multivariate analysis of Bif-1 expression.

Characteristics	Hazard ratio	95% CI	*P *
Bif-1 expression (low versus high)	0.459	0.285–0.739	0.001
Lymph node involvement (yes versus no)	3.231	1.706–6.119	0.002
Distant metastasis (yes versus no)	3.792	1.994–7.303	<0.001
Curative resection (no versus yes)	3.801	2.007–7.199	<0.001

Data calculated using the Cox proportional hazards model. CI: confidence interval.
